# Recent Developments of Circulating Tumor Cell Analysis for Monitoring Cutaneous Melanoma Patients

**DOI:** 10.3390/cancers14040859

**Published:** 2022-02-09

**Authors:** Yoshiaki Shoji, Matias A. Bustos, Rebecca Gross, Dave S. B. Hoon

**Affiliations:** Department of Translational Molecular Medicine, Saint John’s Cancer Institute (SJCI), 2200 Santa Monica Blvd., Santa Monica, CA 90404, USA; ShojiY@jwci.org (Y.S.); Matias.Bustos@providence.org (M.A.B.); Rebecca.Gentry@providence.org (R.G.)

**Keywords:** biomarkers, circulating tumor cells, liquid biopsy, melanoma, molecular diagnostic techniques

## Abstract

**Simple Summary:**

Circulating tumor cells (CTCs) originating from cutaneous melanoma patients have been studied for several decades as surrogates for real-time clinical status and disease outcomes. Here, we will review clinical studies from the last 15 years that assessed CTCs and disease outcomes for melanoma patients. Assessment of multiple molecular melanoma-associated antigen (MAA) markers by quantitative reverse transcriptase-polymerase chain reaction (RT-PCR) was the most common assay allowing for the improvement of assay sensitivity, to address tumor heterogeneity, and to predict patient outcomes. Multicenter studies demonstrate the utility of CTC assays reducing the bias observed in single-center trials. Recent development of CTC enrichment platforms has provided reproducible methods. CTC assessment enables both multiple mRNAs and DNAs genomic profiling. CTC provides specific important translational information on tumor progression, prediction of treatment response, and survival outcomes for cutaneous melanoma patients.

**Abstract:**

Circulating tumor cells (CTCs) have been studied using multiple technical approaches for interrogating various cancers, as they allow for the real-time assessment of tumor progression, disease recurrence, treatment response, and tumor molecular profiling without the need for a tumor tissue biopsy. Here, we will review studies from the last 15 years on the assessment of CTCs in cutaneous melanoma patients in relation to different clinical outcomes. The focus will be on CTC detection in blood samples obtained from cutaneous melanoma patients of different clinical stages and treatments utilizing multiple platforms. Assessment of multiple molecular melanoma-associated antigen (MAA) markers by quantitative reverse transcriptase-polymerase chain reaction (RT-PCR) was the most common assay allowing for the improvement of assay sensitivity, tumor heterogeneity, and to predict patient outcomes. Multicenter studies demonstrate the utility of CTC assays reducing the bias observed in single- center trials. The recent development of CTC enrichment platforms has provided reproducible methods. CTC assessment enables both multiple mRNAs and DNAs genomic aberration profiling. CTC provides specific important translational information on tumor progression, prediction of treatment response, and survival outcomes for cutaneous melanoma patients. The molecular studies on melanoma CTCs have provided and may set standards for other solid tumor CTC analyses.

## 1. Introduction

Cutaneous melanoma arises from the transformation of skin melanocytes to melanoma cells, often related to damaging chronic sunlight UV light exposure [1]. Cutaneous melanoma accounts for the highest skin cancer deaths worldwide with an increasing rate of incidence [2,3], with a poor prognosis in the onset of systemic organ metastasis [4,5]. The incidence rate is increasing in Westernized countries such as the United States, Australia, New Zealand, and Europe [6,7]. Melanoma has a high propensity to metastasize to multiple distant organs if not diagnosed and treated at early stages [2]. Identification of progression at early stages is important to make management decisions to improve melanoma patients’ outcomes.

As primary or metastatic tumors progress, malignant cell(s) are shed into the lymphatic system or peripheral blood system. These cells are referred to as circulating tumor cells (CTCs) and represent a fraction of the tumor cell population [1,8,9,10], as single cells or cell aggregates [11,12,13]. CTCs may have the potential to colonize in tumor-draining lymph nodes and distant organs from the tumor of origin by successfully escaping physical events, host immunity, and organ-specific microenvironmental factors to eventually develop into metastatic colonies [14]. One of the major problems in assessing CTCs in body fluids is their heterogeneity, which often reflects the heterogeneity from the tumor site they originated [15,16,17]. CTCs are important to examine real-time detection of the tumor, tumor recurrence, tumor progression, response to therapy, and assessment of the tumor profile without the need for repetitive biopsies [18]. This is one of the most valuable applications of CTCs, as biopsies are not always feasible and may represent a high-risk procedure for patients.

CTCs derived from solid tumors of different origins can vary in the number released and their metastatic potential. For example, CTCs from cutaneous and acral melanomas have a high propensity to develop into distant organ metastasis [1,19,20]. Contrary to melanoma, CTCs from glioblastoma and hepatocellular carcinoma shed systemically, but rarely metastasize to distant organs [21]. Other solid tumors that have been well studied for CTCs include breast, colon, and prostate cancers [22]. All these tumor types have the potential to metastasize to specific distant organ sites [17]. Melanoma CTCs may spread and establish metastasis to almost all organs in the body, which is a unique property compared to other solid tumors [15].

CTCs have been studied for decades using multiple technical approaches. Many CTC assays have been documented for various types of solid tumors including melanoma; however, each assay has specific limitations. The ultimate utility of CTCs is the real-time detection and accurate correlation with clinical status and/or disease outcomes. Studying cutaneous melanoma CTCs by molecular approaches has been more encouraging recently, in part because of specific transcriptome melanoma-associated genes and gene alteration frequency in melanomas being mapped and related to disease outcomes.

Studies of CTC assessment in relation to melanoma patients’ clinical outcomes until October 2006 are well summarized in a systematic review and meta-analysis by Mocellin et al. [23]. In this article fifty-three studies enrolling 5433 patients were reviewed. CTC detection was based on PCR findings in all but one study, in which immunomagnetic antibody cell enrichment combined with light microscopy was used [24]. CTC identification hinged upon single and multiple tumor markers in 30 (56.6%) and 23 (43.4%) studies, respectively. Among the nine independent variables, the use of multiple markers was the only variable that significantly and independently correlated with higher CTC detection rates (odds ratio (OR), 2.14; 95% confidence interval (CI), 1.1–4.16; *p* = 0.025), which indicates the heterogeneity of melanoma CTCs and the importance of multi-marker assays. Further, CTC status correlated with disease stage (*p* < 0.0001), OS (hazard ratio (HR), 2.42; 95% CI, 1.7–3.45; *p* < 0.0001) and progression-free survival (PFS) (HR, 2.45; 95% CI, 1.78–3.38; *p* < 0.0001), suggesting that CTC analysis offers clinical value in patients with metastatic melanoma.

In this review, we will describe the recent developments of CTC assessment in cutaneous melanoma patients, and the different approaches applied to clinical studies analyzing prognostic information from the last 15 years. The focus will be on the evolution of CTC detection utilizing multiple platforms in the assessment of metastatic melanoma patients of different clinical stages and therapies. In opposition to approaches used frequently in other epithelial cancers, whereby the CTC assessment is primarily based on antibody (Ab) detection, the techniques used for cutaneous melanoma have been focused on direct or indirect assessment of CTCs mRNA melanoma-associated antigens (MAA) biomarkers analysis of peripheral blood mononuclear cells (PBMCs); indirect assays involve CTC enrichment through Ab pull out or fluidic isolation methods followed by molecular assays, whereas direct assessment employed the evaluation of molecular assays without CTC enrichment or Ab staining. The main reason for this strategy is the limited specificity of certain Abs when detecting CTCs in PBMCs [22,25]. Conventional Ab-based approaches are highly dependent on the availability of specific Abs and the binding activity of those to CTCs. The major issue of Ab-based approaches in melanoma is assessing very low levels of CTCs in the peripheral blood, which limits the assay specificity. Further, there are very limited cell surface-specific melanoma-associated antigens (MAAs) and the availability of specific Abs to these antigens.

The most used assay for melanoma CTC assessment in recent years is quantitative reverse transcriptase-polymerase chain reaction (RT-PCR) for messenger RNA (mRNA) with single or multiple gene biomarkers. The MAA genes used must be frequently expressed by melanoma tumors and CTCs and not highly expressed in normal PBMCs. Throughout the years, multiple MAA molecular biomarkers have been developed to address tumor and CTC heterogeneity. During melanoma progression and treatment, the mRNA expression of specific MAA genes becomes heterogeneous within tumors. Tumor heterogeneity and the respective gene expression are the “Achilles’ heel” in melanoma for the patient’s diagnosis as well as treatment. Therefore, the concept of molecular multi-marker analysis of CTCs has become an important approach to improve assay sensitivity, particularly in patients with low CTC levels in the blood. Improved sensitivity and specificity enhance the ability to predict the potential development of metastasis, as well as responses to therapeutic intervention. This has become more relevant now due to the in-depth transcriptome and mutation-based profiling of genes in melanoma and the pathway associated with metastasis as shown by The Cancer Genome Atlas (TCGA; TCGA Research Network: https://www.cancer.gov/tcga accessed on 2 February 2022). Moreover, CTC assessment gains importance in monitoring responses to multiple small molecule inhibitors and monoclonal Ab-based checkpoint inhibitor immunotherapies (CII) approved by the Food Drug Administration (FDA) for melanoma patients [2].

Sensitivity and accuracy in CTC detection are also associated with critical factors during blood collection procedures (including methods of collection, isolation, platforms utilized for purification), data analyses (molecular assay readouts, standardization, reproducibility, specificity of biomarkers for CTCs, CTCs quantification, and results interpretation), and the in-depth quality of the clinical data available (tumor staging, the time of blood draw, and patient follow-up). During blood collection, the type of anticoagulant used as well as the amount of blood utilized for assessment/enrichment (approximately 5–10 mL) is important. There are many types of blood collection tubes and they contain different anticoagulants (e.g., heparin, EDTA, and sodium citrate), but the selection depends on how long after the blood draw the samples are processed and which downstream CTC assays will be performed. Our preference is sodium citrate-containing blood collection tubes because sodium citrate is an anticoagulant that does not interfere with molecular assays and is known to preserve mRNA quality. We also use Cell-Free DNA BCT^®^ tubes (Streck Corporate, NE, USA) which preserve intact CTCs for up to seven days at room temperature. The utility of this approach is in multicenter studies where blood can be shipped to a single centralized laboratory site and assessed several days later.

Due to the development of other types of blood cancer biomarker assays such as cell-free nucleic acids (cfNAs; including cell-free DNA (cfDNA) and cell-free microRNA (cfmiR)), CTCs are now less often used in the clinic for most cancer types, except for metastatic breast, colorectal, and prostate cancer. Nevertheless, CTCs may provide a highly informative assessment of the tumor’s actual systemic spreading, metastatic potential, and overall response to therapy. Since CTCs are often detected in patients with aggressive metastatic melanoma, there is added clinical incentive for the assessment of these types of patients. CTCs remain an important diagnostic factor when monitoring melanoma patients, since these patients can develop metastatic tumors with lethal consequences. Despite the advent of recently approved multiple therapies, the long-term survival outcome remains poor for metastatic diseases. CTCs provide real-time assessment of ongoing systemic metastasis in patients. Hopefully, CTC detection/assessment in cutaneous melanoma patients will help in understanding systemic metastasis in other solid tumors. Furthermore, CTCs can be an alternative for tumor biopsies especially when the tumor is difficult to access, and repetitive monitoring is needed. The development of CTC assay logistics efficiency will enable commercialization, reducing the cost, and may be suitable for large-scale commercial use in the clinical setting for melanoma patients in the future. This review describes the findings of the CTC assays developed in recent years under clinical settings, and the clinical interpretations found in cutaneous melanoma patients.

## 2. Literature Review

**Figure 1:**
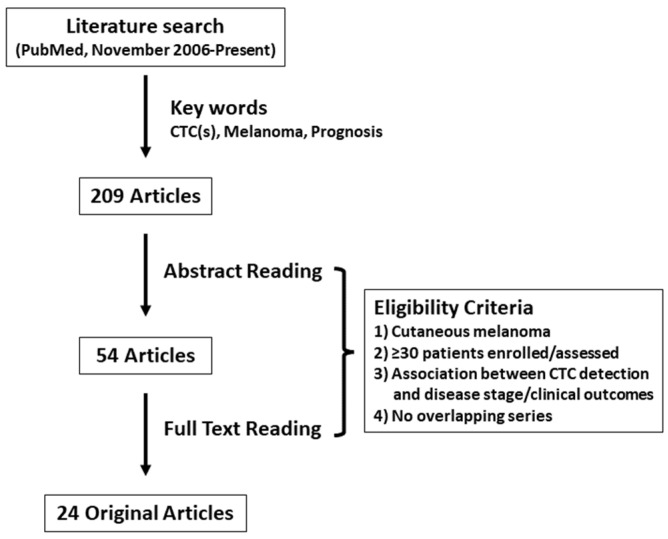
Methodologic flow chart of the review. CTC: circulating tumor cell.

A review of original articles analyzing the prognostic value of CTCs in cutaneous melanoma patients in the last 15 years was performed by searching the PubMed database (https://pubmed.ncbi.nlm.nih.gov/ accessed on 2 February 2022). The search strategy included the following keywords variably combined: “melanoma,” “circulating tumor (melanoma) cell(s)”, and “prognosis”. Original articles after November 2006 were included since the articles published before were discussed in a previous systematic review [23]. Only studies published in peer-reviewed journals were included; data from reviews, letters, and meetings’, abstracts were not considered eligible. The literature search yielded 209 articles. Studies were eligible if disease stage and/or survival data were reported in cutaneous melanoma patients stratified by CTC status (presence/positive versus absence/negative). Articles assessing CTCs for uveal and/or acral melanoma were excluded. Another major restriction criterion was the number of patients enrolled, which was ≥30 in concordance with the previous systematic review [23]. Overlapping and duplicate data sets were excluded. Finally, 24 original articles met all inclusion criteria (Figure 1, Table 1) [26,27,28,29,30,31,32,33,34,35,36,37,38,39,40,41,42,43,44,45,46,47,48,49]. A summary of the 24 articles included in this review is shown in Table 2. Results of five large multicenter trials were reported after November 2006, with a median of 299 patients (148–820) enrolled in those studies. All studies were performed for patients with metastatic disease only or combined with localized disease, except for one multicenter study which analyzed the prognostic impact of CTCs in The American Joint Committee on Cancer (AJCC) stage I and II cutaneous melanoma patients [27]. In 14 studies, CTC analysis was performed for serial bleeds collected during/after treatment. Single-marker RT-PCR for tyrosinase (*TYR*) or melanocyte inducing transcription factor (*MITF*) was performed until 2007, whereas multi-marker RT-PCR became the most common CTC assay from 2008. The most used marker in the multi-marker RT-PCR assays was melan-A (*MLANA*) (n = 12), followed by MAGE family member (*MAGE*) (n = 9) and *TYR* (n = 7). In recent years CELLSEARCH^®^ system (Menarini Silicon Biosystems, Italy), which utilizes immunomagnetic CTC enrichment and fluorescence-based CTC detection, was applied to cutaneous melanoma patients to analyze the prognostic value of CTCs. Two studies analyzed the relationship between CTCs and genomic biomarkers in melanoma patients [28,49]. Most studies (n = 19) determined the predictive impact of CTCs detection on survival outcomes including distant-disease-free survival (DDFS), disease-free survival (DFS), OS, progression-free survival (PFS), and recurrence-free survival (RFS).

## 3. Detection of Melanoma CTCs Using Multi-Marker RT-PCR Assays

The first study demonstrating the utility of multiple molecular MAA CTC markers in cutaneous melanoma patients using a large cohort of multiple clinical stages was reported in 1995 [50]. In this study, four MAA gene markers: *TYR*, melanotransferrin (*MELTF*), melanoma cell adhesion molecule (*MCAM*), and *MAGEA3* were assessed in PBMCs directly from a 10 mL blood sample, without mRNA isolation. The rationale of the multi-marker RT-PCR has been that melanoma cells are heterogeneous and could vary in gene expression during tumor progression or treatment. Within the 119 patients with AJCC stage I–IV disease, CTC detection rates were significantly higher when using four MAA markers rather than a single marker. There was a significant correlation between RT-PCR marker positivity to AJCC stage and disease progression. This initial study demonstrated the potential of direct multi-marker MAA assessment for CTC detection in PBMCs, and from then, multi-marker RT-PCR became one of the most widely used CTC assays in cutaneous melanoma patients.

Most of the RT-PCR studies reported a significant correlation between positive MAA markers and disease status and/or survival, except for the study by Piotr et al. in 2008 [34]. In this study, lymph-fluid and peripheral blood were collected from 107 AJCC stage III cutaneous melanoma patients after radical lymph node dissection. Three MAA gene markers *TYR*, *MLANA*, and universal *MAGE* (*uMAGE*) were detected by multi-marker RT-PCR. Lower estimated twenty-four-month DFS rates were observed for patients with at least one marker (18.9%; 95% CI, 1.4–37.5%; median, 9.9 months) compared to those without any marker (42.1%; 95% CI, 29.7–54.5%; median, 15.3 months) (*p* = 0.04) in lymph-fluid. Although, analysis of the PBMCs did not have additional prognostic value. Reasons for this phenomenon may be the time of blood collection and the volume of the samples collected. Blood samples were collected from the patients between 24 and 48 h after surgery in this study, in which surgical intervention may have affected the CTC analysis. Further, the collected sample volume in this study was 5 mL per patient. Sample volumes ranged from 9–10 mL in the three other studies on CTCs with AJCC stage III melanoma patients included in this review [35,41,48], and all three studies reported a significant correlation between the presence of CTCs and prognosis. Standardization of blood collection and downstream assays are critical factors in CTC analysis, and automated CTC enrichment and detection methodology may provide reliable and reproducible outcomes. This will be further discussed in the “CTC Enrichment and Detection using CELLSEARCH^®^ System” section. Unique features of this study are that they focused on lymph fluid as another source of CTCs, and that they utilized *uMAGE* for CTC detection.

Within the human MAGE family, MAGEA is the most characterized and identified gene in many cancers including melanoma [51]. MAGEA family consists of ≥12 major members that are exclusively expressed in human cancers as well as male germline cells (testis and placenta) [52,53]. Among them, MAGEA1, A2, and A3 were originally well-described antigens in melanoma and cancer originating from the testis [54]. MAGEA1 and 3 have high specificity and expression in various malignancies; however, individual family members are expressed at different frequencies [54]. Therefore, *uMAGEA* primer and probe covering *MAGEA1, -A3, -A5, -A6,* and *-A12* was developed by Miyashiro et al. in 2001 [55]. This was to improve the logistics of assay setup and to obtain a comprehensive multi-marker *MAGEA* MAA family profile in a single assay. In this study, the *uMAGEA* assay increased melanoma detection by 13% compared with the *MAGEA1* assay alone, and by 17% compared with the *MAGEA3* assay alone in melanoma tumors. Further, the *uMAGEA* assay detected CTCs in 24% of melanoma patients’ PBMCs. Although not frequently used in recent studies, *uMAGEA* detection may provide a more practical and sensitive approach for CTC detection compared to a single *MAGEA* family member assay, also requiring less mRNA compared to individual *MAGEA* marker assays.

## 4. Multicenter Trials on CTC Detection for Melanoma Patients

All five multicenter studies included in this article utilized multi-marker RT-PCR for CTC detection. The prognostic impact of CTCs in advanced-stage melanoma was analyzed in all studies, except for the report by Scoggins et al. in 2006 [27], which enrolled AJCC stage I/II patients. This was the largest study included in this review, which applied multi-marker RT-PCR using primers for *MLANA*, pre-melanosome protein (*PMEL*), and *TYR* for CTC detection in PBMCs from serial bleeds collected from 820 patients. One hundred and fifteen (14%) of the patients had evidence of at least one RT-PCR marker present at one of their follow-ups (median follow-up, 37 months). Analysis of individual markers did not differentiate survival outcomes. Although the analysis of baseline blood samples did not show clinical significance in this study, the number of MAA CTC markers expressed revealed that DFS and DDFS were significantly worse for patients with ≥1 marker detected at any point during follow-up compared to patients with negative results (*p* = 0.006 and 0.03, respectively).

In another study by Fusi et al. [35], serial testing for *TYR* and *MLANA* by multi-marker RT-PCR was performed for a subset of patients enrolled in EORTC 18991 phase III trial [56], which evaluated the efficacy and toxicity of pegylated interferon (IFN) versus observation in resected AJCC stage III cutaneous melanoma patients. This study aimed to evaluate the prognostic importance of CTC detection in AJCC stage III melanoma patients after sentinel lymph node (SLN) or regional lymph node dissection. Among 299 patients who underwent final analysis, positive versus negative CTCs at a given time point had no prognostic impact on DDFS. However, Cox time-dependent analysis indicated a significantly higher risk of developing distant metastasis for patients with positive CTCs compared to negative CTCs (HR, 2.23; 95% CI, 1.40–3.55; *p* < 0.001). These two studies indicate the importance of multi-marker RT-PCR analysis in serial bleeds and the value of CTC analysis even in early-stage melanoma.

Two recent multicenter trials included in this review were both reported in 2012, using multi-marker RT-PCR for CTC detection against stage III/IV diseases [40,41]. The study by Hoshimoto et al. [40] analyzed CTCs from patients in a prospective-multicenter international phase III clinical trial to evaluate the efficacy of irradiated whole-cell melanoma vaccine (Canvaxin) (ClinicalTrials.gov identifier: NCT00052156) [57]. After complete metastasectomy, patients rendered disease-free were prospectively randomized to adjuvant therapy with Canvaxin plus Bacille Calmette-Guerin (BCG) versus placebo plus BCG. The blood specimens were collected pre-treatment (n = 244) and during treatment (n = 214) and were evaluated by multi-marker MAA RT-PCR for *MLANA*, *MAGEA3*, and paired box 3 (*PAX3*) CTC mRNA biomarkers. ≥1 CTC MAA biomarkers were detected in 54% of the blood samples from pre-treated patients and in 86% of patients over the first three months of treatment. Median follow-up for the patients was 21.9 months, and during that follow-up, 71% of patients recurred and 48% expired. CTC MAA mRNA levels were not associated with known prognostic factors or treatment arm. In multivariable analysis, CTC status (>0 biomarker versus 0 biomarker) in pre-treatment blood samples were significantly associated with DFS (HR, 1.64; *p* = 0.002) and OS (HR, 1.53; *p* = 0.028). Serial bleeds CTC status was also significantly associated with DFS (HR, 1.91; *p* = 0.02) and OS (HR, 2.57; *p* = 0.012).

In another study by Hoshimoto et al. [41], CTCs were assessed in melanoma patients with SLN metastases in a phase III international multicenter clinical trial [57]. AJCC stage IIIB-D melanoma patients after metastatic lymph node resection are at high risk of developing distant organ metastasis [58]. However, there were no specific blood biomarkers to determine which patients will develop metastasis and should be triaged onto adjuvant therapy. Blood specimens were collected from patients with melanoma (n = 331) who were clinically disease-free after having a complete lymph node dissection (CLND) before entering onto a randomized adjuvant melanoma cell vaccine plus BCG versus placebo plus BCG trial from 30 centers. Blood was assessed by a multi-marker RT-PCR assay using *MLANA*, *MAGEA3*, and beta-1,4-N-acetyl-galactosaminyltransferase 1 (*B4GALNT1*) as MAA markers. Individual CTC biomarker detection rates ranged from 13.4–17.5%. Cox regression analyses were used to evaluate the prognostic significance of CTC status for disease recurrence and disease-specific survival (DSS). There was no association of CTC status (≤1 biomarker versus ≥2 biomarkers) with known clinical or pathologic prognostic variables. However, ≥2 positive CTC MAA biomarkers were significantly associated with reduced DDFS (HR, 2.13; *p* = 0.009), RFS (HR, 1.70; *p* = 0.046), and DSS (HR, 1.88; *p* = 0.043) in multivariable analyses. The CTC MAA marker *B4GALNT1* used in this study was originally described by Kuo et al. in 1998 [59]. B4GALNT1 is involved in the synthesis of GM2 and GD2, key melanoma cell surface glycosphingolipids [60,61], which is frequently found in advanced-stage melanomas and in patients showing more aggressive melanoma tumors, whereas normal donor PBMCs were negative for *B4GALNT1* mRNA expression. *B4GALNT1* was used in four multi-marker RT-PCR studies included in this review [28,37,41,49], and all four studies reported a significant correlation between CTC detection and survival outcomes for metastatic melanoma patients.

The above multicenter studies demonstrate the utility of CTC MAA multi-marker RT-PCR assays in patients who rendered disease-free for the monitoring of recurrent distant metastatic diseases, reducing the bias observed in single-center trials.

## 5. CTC Detection in Association with Treatment Response

Since November 2006, five studies reported a correlation between CTC detection and treatment responses. Only three of these studies reported the impact of CTCs detection in serial blood samples from metastatic melanoma patients by multi-marker RT-PCR. The study by Koyanagi et al. [37] was designed to assess whether CTC detection could be used as a surrogate for tumor progression and treatment response in metastatic melanoma patients. Subjects for this study were enrolled in a prospective multicenter phase II clinical trial of concurrent decrescendo bio-chemotherapy (BC) combined with maintenance biotherapy (mBT) for AJCC stage IV melanoma patients [62]. The BC regimen contained cisplatin, dacarbazine, vinblastine, interleukin (IL)-2, IFNα2b, and granulocyte macrophage colony-staining factor (GM-CSF), and the treatment was repeated every 21 days. The mBT regimen was a 28-day cycle of low-dose IL-2 and GM-CSF, which included intermittent pulses of high-dose decrescendo IL-2. Blood samples were collected from 87 AJCC stage IV melanoma patients before and during induction of BC and mBT. Expression of five MAA CTC mRNA biomarkers: *B4GALNT1*, *MAGEA3*, *MITF*, *MLANA*, and *PAX3* were assessed by RT-PCR and correlated with disease outcomes. The number of positive CTCs MAAs decreased overall during BC treatment (*p* < 0.0001). CTC biomarker detection after two cycles of BC was correlated with treatment response (*p* = 0.005) and OS (*p* = 0.001), whereas an increase in the number of CTC mRNA biomarkers was associated with poor treatment response (*p* = 0.006) and OS (*p* < 0.0001). Multivariate analyses using a Cox proportional hazards model identified the change in CTC biomarkers after two cycles of BC as an independent factor for disease progression (risk ratio (RR), 12.6; 95% CI, 4.78–33.4; *p* < 0.0001) and OS (RR, 6.11; 95% CI, 2.37–15.7; *p* = 0.0005).

The use of *MITF* as a molecular marker to detect cutaneous melanoma CTCs and its clinical significance was initially described by Koyanagi et al. under clinical settings in 2006 [29]. MITF is important in melanocyte development and melanoma growth and was therefore assessed by RT-PCR in 148 patients with AJCC stage I–IV cutaneous melanoma in this study. The rate of *MITF* detection was higher with increasing AJCC stage (*p* < 0.0001). *MITF* detection after BC was associated with a significantly lower RFS (*p* < 0.0001) and OS (*p* = 0.001). Furthermore, *MITF* detection after BC was an independent prognostic factor for RFS (RR, 5.63; *p* = 0.0004) and OS (RR, 5.36; *p* = 0.005).

In another study by Reid et al. in 2013, multi-marker RT-PCR was performed for *MLANA*, ATP binding cassette subfamily B member 5 (*ABCB5*), *MCAM*, transforming growth factor-beta 2 (*TGFB2*), and *PAX3*, in 230 patients with AJCC stage I–IV melanoma. The multi-marker detection was correlated with clinical outcomes and treatment responses. Two markers, *MLANA* (45%, *p* = 0.001) and *ABCB5* (49%, *p* = 0.031), had the greatest prognostic values and were identified as statistically significant factors among patients who experienced disease recurrence within the study period. In the same study, thirty-four patients (15%) received nonsurgical treatments, which included radiotherapy (n = 29), IFNα2b (n = 5), limb infusion (n = 1), vaccine (n = 4), chemotherapy (n = 9) and radio frequency ablation (n = 1). 43% (n = 17) of the samples obtained from patients with negative treatment outcomes expressed *MCAM*, while only 9% (n = 2) of the samples taken from patients with a positive outcome expressed *MCAM* (*p* = 0.006).

A recent study that analyzed CTCs in metastatic melanoma patients receiving CII [49] will be further discussed in the “CTC and genomic biomarkers” section. In summary, CTC-derived MAA biomarkers may have the utility for monitoring response to various treatments including BC, mBT, and CII therapies in melanoma patients.

## 6. CTC Enrichment and Detection Using CELLSEARCH^®^ System

Although single-center and multicenter studies utilizing multi-marker RT-PCR have reported a robust relationship between CTC detection and clinical outcomes, there is still a lack of standardization of methodologies and unification of CTC biomarkers. CELLSEARCH^®^ System is a semi-automated methodology approved by the FDA for analyzing CTCs in metastatic breast, colon, and prostate cancers. In this system, ferrofluids coated with CD146 Abs were used to immunomagnetically enrich CTCs, and a fluorescent labeled high molecular weight melanoma-associated antigen (HMW-MAA, chondroitin sulfate proteoglycan 4) specific Ab was utilized for CTC detection in melanoma, as well as CD45 and CD34 Abs for exclusion of white blood cells and endothelial cells. Six recent studies on melanoma CTCs [39,42,45,46,47,48] have utilized this technology. The initial study using the CELLSEARCH^®^ System for melanoma CTC detection under clinical settings was reported by Rao et al. in 2010 [39]. In this study, a retrospective analysis compared the CTC numbers and OS in 79 blood samples obtained from five unresectable stage III and 39 stage IV melanoma patients. The number of CTCs ranged from 0 to 8042 per 7.5 mL of blood collected from each melanoma patient, whereas ≤1 CTC per 7.5 mL of blood collected was detected in all 55 healthy donors. Patients with ≥2 CTCs had a significantly shorter OS compared to patients with <1 CTC (median OS, 2.0 and 12.1 months, respectively; HR, 3.2; 95% CI, 1.6–6.5; *p* = 0.001).

In the most recent and largest study utilizing CELLSEARCH^®^ Circulating Melanoma Cell Assay by Lucci et al. [48], CTCs were analyzed for 243 patients with AJCC stage III melanoma at their first clinic visit. CTC detection was not associated with substage or primary tumor characteristics; however, multivariable analysis demonstrated that the detection of ≥1 CTC at baseline was significantly associated with a decreased 6-months RFS (HR, 3.62; 95% CI, 1.78–7.36; *p* < 0.0001) and 54-months RFS (HR, 1.69; 95% CI, 1.13–2.54; *p* = 0.01). The CELLSEARCH^®^ platform is standardized, and therefore provides reproducible results, and can easily be assimilated by other centers. Limitations are the CTC enrichment and capture method, since lack of CD146 and HMW-MAA expression in certain CTCs released by melanoma tumors may result in missing CTC detection [63]. The expression of CD146 and HMW-MAA in various normal non-melanoma cells may result in false positives; therefore, it is not recommended to be used alone as a melanoma CTC molecular biomarker. An important issue for these types of assays is the availability of highly specific melanoma CTC MAA cell-surface Abs. As the profiling of melanoma cell surface proteins and carbohydrates advances, new MAA cell-surface Abs may be identified and employed in CTC detection. Further development and improvements may be needed for a more sensitive and specific platform.

## 7. CTC and Genomic Biomarkers

Several previous studies have separately shown the utility of CTCs and cfNAs found in the blood of cancer patients, however, the combination of these two blood biomarkers has rarely been reported. In the study by Koyanagi et al. [28], matched pairs of PBMCs and serum specimens were obtained from 50 AJCC stage IV melanoma patients before administration of BC. Three CTC MAA markers *B4GALNT1*, *MAGEA3*, and *MLANA* were analyzed by multi-marker RT-PCR. Further, serum was analyzed for two methylated MAA DNA markers: Ras association domain family member 1A (*RASSF1A*) and retinoic acid receptor beta 2 (*RARB2*) as previously described [64]. CTCs were detected in 13 of the 15 (86%) patients with serum tumor-related methylated DNA and only in 13 of 35 (37%) patients without methylated DNA (*p* = 0.001). The number of CTC MAA markers detected significantly correlated with methylated cfDNA detection (*p* = 0.008). CTC and methylated cfDNA were also significantly correlated with poor response to BC (*p* = 0.02), shorter time to progression (*p* = 0.009), and worse OS (*p* = 0.02).

In the latest study by Lin et al. [49], CTCs were enriched and assessed for both MAA mRNA and DNA biomarkers to explore their utility in monitoring patients receiving CII alone or in combination with targeted therapies. Blood samples (n = 213) were collected prospectively from 75 AJCC stage III/IV melanoma patients before and during CII treatment. CTCs were enriched using a microfluidics-based CTC platform, ClearCell^®^ FX system (Clearbridge BioMedics, Singapore) [65]. The ClearCell^®^ FX system is an automated CTC enrichment system driven by a microfluidic biochip to isolate CTCs based on size, deformability, and inertia. In this study, CTCs were profiled for five established MAA mRNA biomarkers: *B4GALNT1*, *MAGEA3*, *MLANA*, *PAX-3*, and TRNA-Pro (anticodon AGG) 2-6 (*TRP-AGG2-6*). B-Raf proto-oncogene, serine/threonine kinase (*BRAF*) *V600E* single-nucleotide variant (SNV) was assessed by the RainDrop^®^ Digital Droplet PCR *BRAF V600E* Assay (Bio-Rad, Hercules, CA, USA). CTCs were detected in 88% (188 of 213) of the blood samples collected from 75 AJCC stage III/IV melanoma patients during CII treatment. CTC-derived biomarkers and clinical variables analyzed using classification and regression tree (CART) analysis revealed that a combination of molecular MAA markers and serum lactate dehydrogenase (sLDH) was indicative of clinical outcomes for patients with AJCC stage IV melanoma (n = 52). The incorporation of *BRAF V600E* SNV with MAA mRNA profiling increased the overall CTC-detection capability by 57% compared to MAA mRNA profiling alone. This study demonstrated that microfluidics-based CTC enrichment successfully isolated CTCs from PBMCs. The drawbacks of CTCs consist of immediate blood processing, and the purity of CTCs needed from blood PBMCs using microfluidic separation.

These studies indicate that the combination of CTCs isolation with MAA mRNA and cfNA genomic biomarker analysis may have the potential to improve disease detection rate and predict melanoma patients’ outcomes. Further development of multi-omic blood biomarker platforms including those approaches involving single-cell molecular analyses [66] may be more informative and benefit cutaneous melanoma patients’ monitoring.

## 8. Other Prognostic Biomarker for Cutaneous Melanoma Patients

CfNA (cfDNA and cfmiRs) and circulating extracellular vesicles (EVs) have also emerged as promising biomarkers in solid tumors. Specific cfmiRs in cutaneous melanoma patients may also potentially allow for the assessment of melanoma patients developing systemic organ metastasis, and to predict treatment responses to checkpoint inhibitor immunotherapy [67]. Melanoma-derived circulating EVs have been shown to play an important role in tumor progression and have increased attention in relation to disease progression and treatment responses [68,69]. However, further development, standardization of isolation/detection methods, and validation are needed to be applied in clinical settings.

CfDNA released by tumor cells (circulating tumor DNA, ctDNA) has also emerged as a promising biomarker in cutaneous melanoma, as a source of information for specific gene alterations. The most common allelic variants (AV) in cutaneous melanoma tissues include *BRAF*, neuroblastoma RAS viral oncogene homolog (*NRAS*), mitogen-activated protein kinase kinase 1 (*MAP2K1*), and proto-oncogene c-KIT (*KIT*). Among them, the most common allelic variant at a specific nucleotide is the *BRAF* V600 SNV, which accounts for 40–50% of cutaneous melanoma patients [70]. *BRAF* V600E SNV has been extensively studied in cutaneous melanoma patients, as the status is associated with clinical outcomes and response to targeted therapies [1]. The detection of *BRAF* V600E SNV in cfDNA released by living, dying, or dead tumor cells using patient plasma or serum has also been associated with tumor burden, minimal residual disease, treatment response, and clonal evolution [71].

The first study which analyzed *BRAF* AV in ctDNA of cutaneous melanoma patients was reported in 2009 by Shinozaki et al. [72]. In this study, RT-PCR was utilized to detect *BRAF* AV in 103 plasma samples from AJCC stage I–IV melanoma patients, including 48 AJCC stage IV patients who received BC. CtDNA *BRAF* AV was detected in a total of 38 (37%) patients enrolled in this study. The post-BC ctDNA *BRAF* AV detection rate was significantly higher in non-responders compared to responders (*p* = 0.02), and further, *BRAF* AV status (positive/negative) associated with significantly worse OS (median OS, 13.0 and 30.6 months, respectively; *p* = 0.039). This initial study demonstrated that *BRAF* AV in ctDNA is detectable, and the clinical importance of ctDNA *BRAF* AV monitoring to predict treatment responses and survival for cutaneous melanoma patients.

Recently, a large clinical study analyzed ctDNA isolated from plasma samples obtained at baseline from 556 patients with *BRAF V600E/K* SNV positive melanoma who were enrolled in BREAK-2 [73], BREAK-3 [74], BREAK-MB [75], and METRIC [76] studies. *BRAF* V600E/K SNV for ctDNA was assessed using emulsion PCR on magnetic beads and flow cytometry [77]. *BRAF* V600E/K SNV was detectable in 76% and 81% of patients with *BRAF V600E/K* SNV-positive tumor tissues, respectively. Patients negative for *BRAF* SNV in ctDNA had longer PFS and had higher response rates to dabrafenib and trametinib compared to patients with *BRAF SNV,* except for the cohort enrolled in the MREAK-MB study where the response rate to dabrafenib was 44% and 43% for patients with or without ctDNA *BRAF* SNV, respectively. Multivariate analysis including LDH, ECOG status, disease stage, number of metastatic sites, and visceral disease as covariates indicated that ctDNA *BRAF* V600E SNV was an independent predictor of PFS in the BREAK-2 and -3 studies, and an independent predictor of OS in BREAK-3 study. In another recent large clinical study by Mahrukh et al. [78], ctDNA *BRAF* V600E SNV was analyzed in 383 baseline and 262 on-treatment plasma samples for patients enrolled in COMBI-d [79] and COMBI-MB [80] studies using droplet digital PCR assays to evaluate whether ctDNA could predict survival outcomes for advanced cutaneous melanoma patients treated with dabrafenib or dabrafenib plus trametinib. In this study, ctDNA was detected in a total of 354 (92%) of the baseline samples. In a subset of patients enrolled in COMBI-d, baseline ctDNA *BRAF* V600E SNV status was significantly associated with worse OS (HR, 1.13; 95% CI, 1.09–1.18; *p* < 0.0001), and a ctDNA cutoff point of 64 copies per mL stratified PFS and OS for the patients. Further, undetectable ctDNA on-treatment was significantly associated with extended PFS and OS, particularly in patients with elevated LDH (PFS; HR, 1.99; 95% CI, 1.08–3.64; *p* = 0.027 and OS; HR, 2.38; 95% CI, 1.24–4.54; *p* = 0.009, respectively). These two studies have verified the clinical impact of ctDNA *BRAF* V600E SNV detection for cutaneous melanoma patients.

CtDNA analysis has been extensively shown to be a reliable tool in predicting patient outcomes and monitoring treatment responses in patients with solid tumors. Compared to CTCs, ctDNA has the added advantage of being easily collected and stored for analysis at a later date, which may provide clinical benefits [81]. In recent years, several technical advances including droplet digital PCR (ddPCR) and next-generation sequencing (NGS) have extended the application and improved the sensitivity of ctDNA assays in the evaluation of therapy responses [82,83]. Improved sensitivity of ddPCR as compared to conventional PCR methods has enabled the detection of low levels of ctDNA and gene alteration frequency with lower costs than NGS approaches [84]. NGS techniques have overcome the limitation of PCR assays, in which the screening was limited to the most frequent known gene alterations. Targeted-sequencing panels in combination with PCR have further enabled to improve the assay sensitivity and specificity [85]. In a study by Lin et al. [86], a 54-cancer gene panel digital NGS assay was utilized to analyze ctDNA SNVs and copy number amplification for monitoring disease in a total of 142 blood samples from AJCC stage I–IV cutaneous melanoma patients. In this study, ctDNA SNVs were detected in 75% of the patients. The number of unique SNVs detected and the total cumulative SNV variant allele fraction were significantly associated with worse OS (*p* = 0.026 and *p* = 0.049, respectively) in AJCC stage IV patients. CtDNA SNV levels significantly correlated with tumor burden (*p* = 0.019), and the detection of ctDNA SNVs enabled earlier detection of recurrence compared with radiologic imaging (*p* < 0.001). This study demonstrated the significant utility of targeted-sequencing assay, for earlier detection of recurrence and to predict disease progression in cutaneous melanoma patients.

One of the main limitations for ctDNA assays is the origin, and whether the ctDNA is actively released from live cells or derived from cells that have undergone apoptosis/necrosis. CtDNA can also be derived from CTCs that have undergone apoptosis/necrosis. Thus, ctDNA may be more representative of CTCs that are abundant in the systemic blood of patients. A recent study has shown that ctDNA detection may not be efficient to detect disease progression in melanoma patients compared to standard positron emission tomography imaging [87]. Further, prior knowledge of the hotspot SNV is required for this ctDNA-based monitoring, as it has been shown that the monitoring is unsuccessful for melanoma patients that lack *BRAF* V600E SNV [88]. The use of ctDNA analysis may be limited for gene alterations with low frequency and detectability.

Serial bleed monitoring of CTC, cfNA, and EV have their own set of advantages and limitations; however, they provide diagnostic and prognostic information that may improve patient management and outcomes when combined.

## 9. Conclusions

Studies in the last 15 years have demonstrated that CTCs in cutaneous melanoma patients are detectable and have potential clinical utility. The application of different assays utilized in previous years coincided with the evolution of techniques and melanoma therapies. Analysis of MAA biomarkers by RT-PCR suggests that many CTCs are present in melanoma patients’ peripheral blood. Different levels of expression among MAA biomarkers during follow-up demonstrate the heterogeneity of CTCs. The utility of multi-marker MAA RT-PCR provides clinical information which correlates to disease outcomes in treated and non-treated patients. Multicenter trials reduced the bias observed in single-center trials. The development of novel CTC enrichment methods has provided standardized and reproducible results. In addition, CTC enrichment enables the assessment of both multiple MAA mRNAs and DNA gene alteration profiling. Further multicenter clinical studies may be needed to determine the value of CTCs enrichment/assessment and cfNAs monitoring alone or in combination with CTCs, for predicting patient outcomes, monitoring diseases, and selecting patients who will benefit from aggressive treatment or surveillance.

As newer technologies for CTC enrichment/detection become available, the field of CTC analysis will expand. The recent development of molecular/proteomic profiling may improve the information gained from CTCs, and the potential clinical utility of CTC assessment for cutaneous melanoma patients.

## Figures and Tables

**Table 1:** Cutaneous melanoma CTC studies included in this review.

Author	Year	Multicenter	N	Blood Draw	CTC Enrichment/ Detection	CTC Markers	AJCC Stage	Outcomes Related to CTC Detection
G Palmieri et al. [26]	2006	No	149	Multiple	MM PCR	MELTF, MLANA, TYR	I–III	Recurrence, Survival (DFS, OS)
CR Scoggins et al. [27]	2006	Yes	820	Multiple	MM PCR	MAGEA3, MLANA, PMEL, TYR	I, II	Survival (DDFS, DFS)
K Koyanagi et al. [28]	2006	No	50	Single	MM PCR	B4GALNT1, MAGEA3, MLANA	IV	Survival (OS, PFS), Treatment response
K Koyanagi et al. [29]	2006	No	148	Multiple	SM PCR	MITF	I–IV	AJCC Stage, Survival (OS, RFS)
E Carrillo et al. [30]	2006	No	58	Single	SM PCR	TYR	I-IV	AJCC Stage, Recurrence, Survival (OS)
P Quaglino et al. [31]	2007	No	200	Multiple	SM PCR	TYR	IV	Progression, Survival (OS, PFS), Treatment response
C Visús et al. [32]	2007	No	114	Single	SM PCR	TYR	I–IV	AJCC Stage, Survival (DFS, OS, PFS)
P Arenberger et al. [33]	2008	No	65	Multiple	MM PCR	MLANA, PMEL, MAGEA3, MIA, TYR	II–III	Progression
P Rutkowski et al. [34]	2008	No	107	Single	MM PCR	MLANA, TYR, uMAGE	III	None
A Fusi et al. [35]	2009	Yes	299	Multiple	MM PCR	MLANA, TYR	III	Progression
S Gkalpakiotis et al. [36]	2010	No	65	Multiple	MM PCR	MAGEA3, MIA, MLANA, PMEL, TYR	II, III	Progression, Survival (DFS)
K Koyanagi et al. [37]	2010	Yes	87	Multiple	MM PCR	B4GALNT1, MAGEA3, MITF, MLANA, PAX3,	IV	Progression, Survival (OS), Treatment response
I Samija et al. [38]	2010	No	201	Single	MM PCR	MITF, TYR	I-IV	Survival (OS, PFS)
C Rao et al. [39]	2011	No	44	Single	CELLSEARCH^®^	CD146, HMW-MAA	III, IV	Survival (OS)
S Hoshimoto et al. [40]	2012	Yes	244	Multiple	MM PCR	MAGEA3, MLANA, PAX3	IV	Survival (DFS, OS)
S Hoshimoto et al. [41]	2012	Yes	331	Single	MM PCR	B4GALNT1, MAGEA3, MLANA	III	Survival (DDFS, DSS, RFS)
L Khoja et al. [42]	2013	No	101	Multiple	CELLSEARCH^®^	CD146, HMW-MAA	IV	Survival (OS)
AL Reid et al. [43]	2013	No	230	Multiple	MM PCR	ABCB5, MCAM, MLANA, PAX3, TGFB2, PAX3	0–IV	Recurrence, Treatment response
ES Gray et al. [44]	2015	No	56	Multiple	Flow cytometry	ABCB5, CD146, CD271, HMW-MAA, RANK	I–IV	Survival (PFS)
CL Roland et al. [45]	2015	No	89	Single	CELLSEARCH^®^	CD146, HMW-MAA	I–IV	AJCC Stage
J Li et al. [46]	2018	No	100	Multiple	CELLSEARCH^®^	CD146, HMW-MAA	I–IV	AJCC Stage, Survival (OS, PFS, DSS)
CS Hall et al. [47]	2018	No	93	Single	CELLSEARCH^®^	CD146, HMW-MAA	IV	Survival (PFS)
A Lucci et al. [48]	2020	No	243	Single	CELLSEARCH^®^	CD146, HMW-MAA	III	Survival (RFS)
SY Lin et al. [49]	2020	No	75	Multiple	ClearCell^®^ FX/MM PCR	B4GALNT1, MAGEA3, MLANA, PAX3, TRP-AGG2-6	III, IV	Survival (DFS, OS), Treatment response

CTC: circulating tumor cell, N: number of patients, AJCC: The American Joint Committee on Cancer, MM PCR: Multi-marker reverse transcriptase-polymerase chain reaction, SM PCR: Single-marker reverse transcriptase-polymerase chain reaction, DFS: disease-free survival, OS: overall survival, DDFS: distant-disease free survival, PFS: progression-free survival, RFS: recurrence-free survival, DSS: disease-specific survival, HMW-MAA: high molecular weight melanoma-associated antigen, ABCB5: ATP binding cassette subfamily B member 5, RANK: receptor activator of nuclear factor kappa B. Official gene symbols approved by the HGNC (https://www.genenames.org/, accessed on 2 February 2022) are used for gene names.

**Table 2:** Summary of the melanoma CTC studies included in this review.

Variables		N
Study design	Single-center	19
	Multicenter	5
Disease status	Localized	1
	Metastatic	12
	Both	11
Blood draw	Single	10
	Multiple	14
CTC enrichment	CELLSEARCH^®^	6
	ClearCell^®^ FX	1
	None	17
CTC detection	Multi-marker RT-PCR	13
	Single-marker RT-PCR	4
	CellSearch	6
	Flow cytometry	1
RT-PCR mRNA markers	*MLANA*	12
	*TYR*	10
	*MAGE*	9
	*PAX3*	4
	*PMEL*	3
	*MITF*	3
	*MIA*	2
	*B4GALNT1*	2
Outcomes	Survival	19
	AJCC Stage	5
	Progression	5
	Treatment response	5
	Recurrence	3

CTC: circulating tumor cell, N: number of studies, RT-PCR: reverse transcriptase-polymerase chain reaction, mRNA: messenger RNA, AJCC: The American Joint Committee on Cancer. Official gene symbols approved by the HGNC (https://www.genenames.org/, accessed on 2 February 2022) are used for gene names.
